# Supplementary material for: Construction of the experimental rat model of gestational diabetes
Source: PLoS One. 2022 Sep 15;17(9):e0273703. doi: 10.1371/journal.pone.0273703 (PMC9477341; doi:10.1371/journal.pone.0273703)
Supplement: S1 File — (DOCX) [file pone.0273703.s001.docx]

**Original images for blots and gels**

| **Protien** | **Original blot** | **Minimally adjusted images** |
| --- | --- | --- |
| **β-actin** | 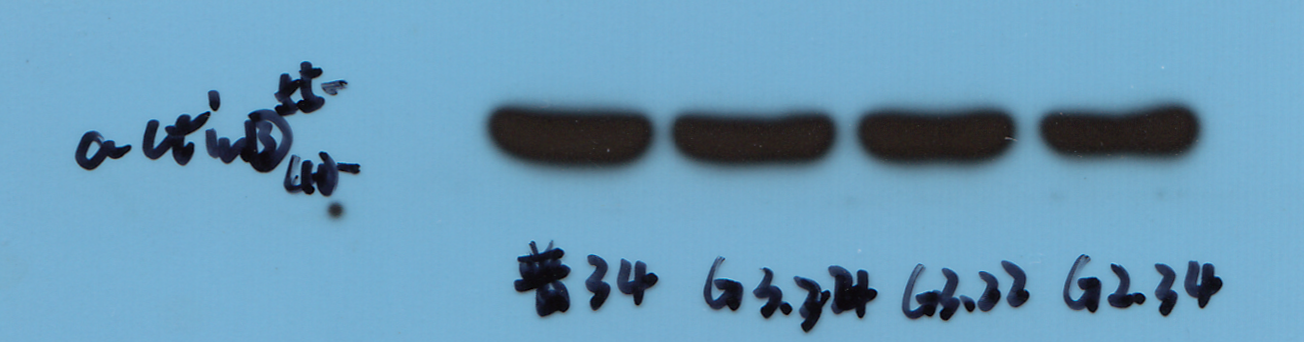 | 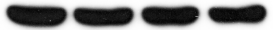 |
|  | 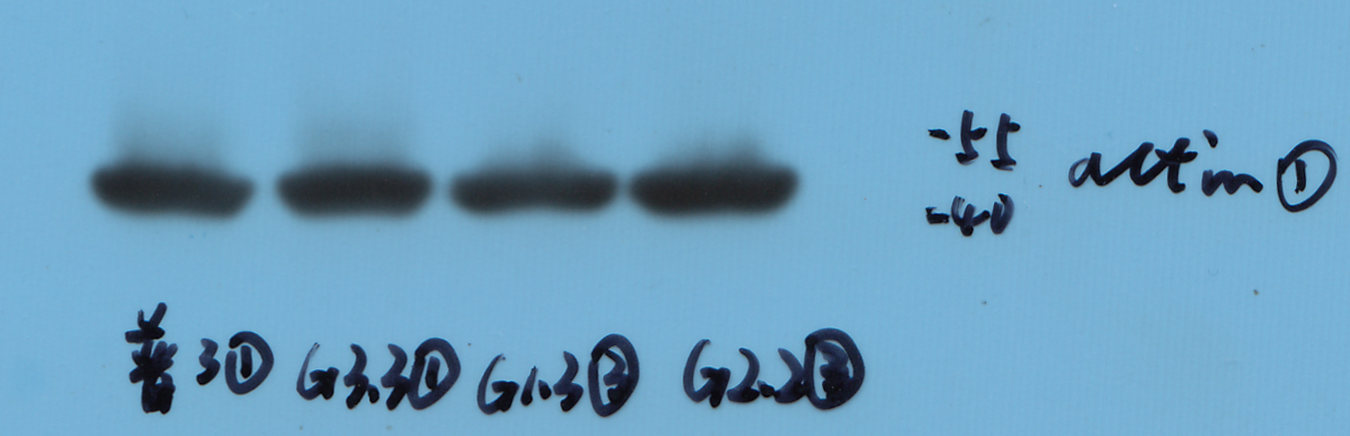 | 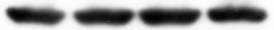 |
|  | 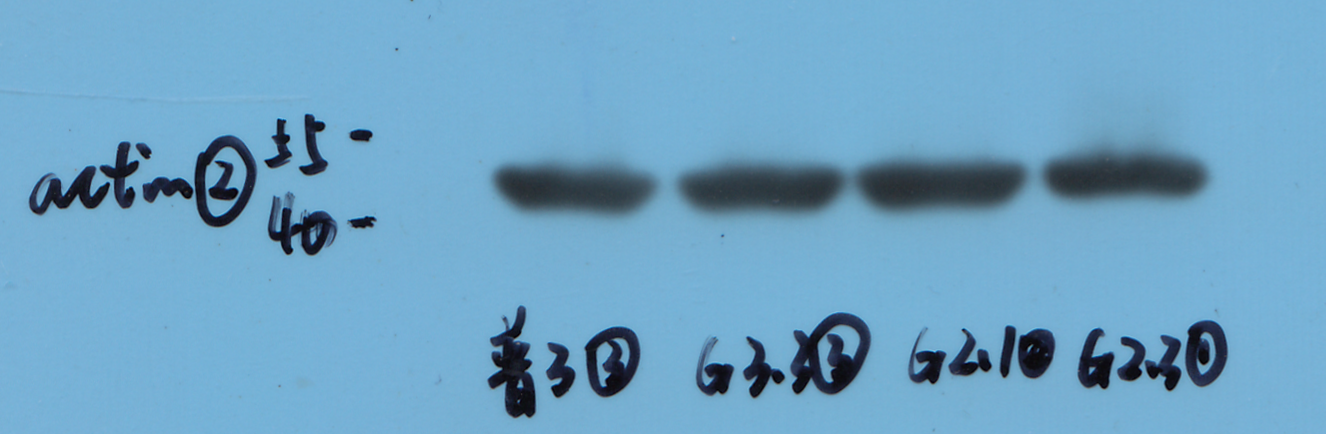 | 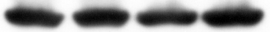 |
| **GLUT1** | 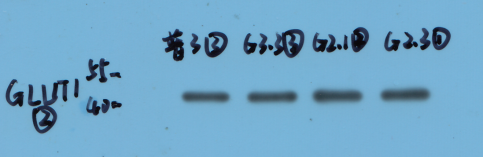 | 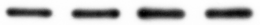 |
|  | 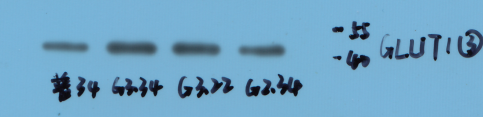 | 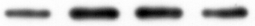 |
|  | 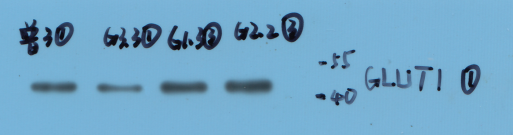 | 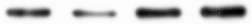 |
| **GLUT3** | 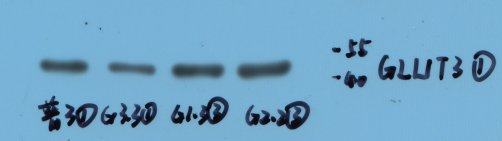 | 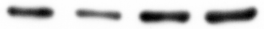 |
|  | 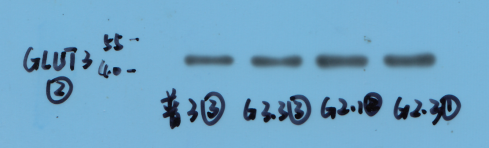 | 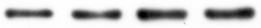 |
|  | 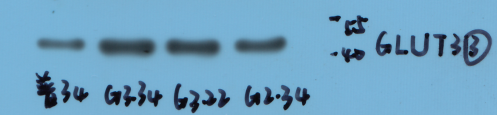 | 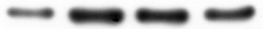 |
